# A cell-based high-throughput screening assay system for inhibitor compounds of antigen presentation by HLA class II molecule

**DOI:** 10.1038/s41598-017-07080-4

**Published:** 2017-07-28

**Authors:** Nobuo Watanabe, Yusuke Suzuki, Takahisa Yonezu, Yuki Nakagawa, Takashi Shiina, Noriaki Hirayama, Sadaki Inokuchi, Shigeaki Inoue

**Affiliations:** 1 Department of Emergency and Critical Care Medicine, 143 Shimokasuya, Isehara, Kanagawa, 259-1193 Japan; 2 Department of Molecular Life Science, 143 Shimokasuya, Isehara, Kanagawa, 259-1193 Japan; 3 Institute of Advanced Biosciences, Tokai University School of Medicine, 143 Shimokasuya, Isehara, Kanagawa, 259-1193 Japan

## Abstract

A number of autoimmune diseases are associated with the genotypes of human leukocyte antigen class II (HLA), some of which present peptides derived from self-proteins, resulting in clonal expansion of self-reactive T cells. Therefore, selective inhibition of self-peptide loading onto such disease-associated HLA could ameliorate the diseases. To effectively identify such compounds, in this study, we established, for the first time, a cell- and 96-well microplate-based high-throughput screening system for inhibitors of antigen presentation. A panel of DRB1 genes plus DRA*01:01 gene were expressed in HEK293T cells and in 3T3 cells, and their binding with biotinylated known self-antigen peptides was measured by flow cytometry. HLA-DR1 (DRB1*01:01) and DR15 (DRB1*15:01) showed a high affinity with myelin basic protein peptide (MBP83-98). Therefore, in 96-well plate wells, MBP83-99 was allowed to bind to DR1 or DR15 on 3T3 cells in competition with a test compound, and the HLA-bound peptide was detected by streptavidin-conjugated β-galactosidase, thereby identifying inhibitor compounds for rheumatoid arthritis or multiple sclerosis. Our assay system has a potential for broad applications, including designing peptide vaccines.

## Introduction

Human leukocyte antigen class II (HLA) molecules are expressed on the surface of antigen presenting cells (APCs), including dendritic cells and B cells, and present peptides derived from captured foreign protein antigens for the surveillance of CD4^+^ T cells^1, 2^. On the HLA molecules, antigen-derived peptides are immobilised in the peptide-binding groove that is composed of α- and β-chains^1^. HLA class II constitutes three classes, namely, DR, DQ, and DP. While the DNA sequences for α-chain are almost conserved in each class, those for β-chain present polymorphism, resulting in the diversity and specificity of peptide binding. In the DR class of HLA (HLA-DR), the α-chain is exclusively coded by DRA*01:01 allele whereas allelic variants of the β-chain (DRB) exceed 1700^3^.

An array of autoimmune diseases, including rheumatoid arthritis (RA) and multiple sclerosis (MS), are associated with particular alleles of HLA-DRB1^1, 3^. Accumulating data demonstrated that some autoimmune disease-associated HLA-DR molecules display peptides derived from self-antigens, which consequently induces clonal expansion of the HLA-restricted antigen-specific CD4^+^ T cell. For instance, HLA-DRB1*01:01 and DRB1*04:01 alleles are associated with RA, and those gene-derived HLA molecules, namely, DR1 and DR4, respectively, present peptide from type II collagen (CII263-272)^4, 5^. On the other hand, HLA-DRB1*15:01 is linked to MS, and DR15 molecules present a myelin basic protein-derived peptide (MBP83-99)^6, 7^. Over the past decade, increasing numbers of peptides displayed on various autoimmune disease-associated HLA-DRB1 molecules have been identified. As such, selective blockade of the peptide loading onto disease-associated HLA could potentially suppress the progression of the autoimmune disease without affecting immune functions mediated by other HLAs. To this end, small-molecule compounds capable of blocking peptide loading onto HLA have been developed as potential therapeutics for MS^7, 8^, RA^9, 10^, and thyroiditis^11^. In these studies, screening and initial verification of molecular interaction of the compounds were carried out in a cell-free assay system using recombinant HLA molecules^9, 11^.

Because HLA is an α/β heterodimeric glycosylated membrane protein, conventional *E.* *coli* expression systems are not applicable for the protein production. Various recombinant HLA proteins were engineered and expressed in yeast^12^ or insect cells^9, 13, 14^. Using these HLA molecules, affinity and specificity between particular antigen peptides and HLA were evaluated, and, in combination with 96-well or 386-well plates and a plate reader, cell-free high-throughput screening systems for compounds that can inhibit or even enhance peptide loading onto HLA molecules have been developed^12, 15–17^. To the best of our knowledge, however, there is no substantial report on antigen binding assay conducted on HLA-transfected cultured cells in 96- or 385-well plates and revealed by using a plate reader.

Expression of functional HLA molecules in non-APCs in terms of peptide presentation capacity has also been challenged by ways of transfection with DRA and DRB genes. Although HLA molecules are in general unstable without accessory chaperone molecules such as CD74 and HLA-DM and/or occupancy of antigen peptides or class II-associated invariant chain peptide (CLIP)^18^, successful cases of cell-surface expression have been reported^19–21^. Nevertheless, assessment of the binding between antigen peptides and HLA molecules on these transfected cells was exclusively conducted by FACS analysis^17, 21^ or by monitoring the proliferation of antigen-specific T cell hybridomas^17, 22^. To establish a high throughput screening system of inhibitor compounds of peptide loading onto HLA molecules in cultured cells, fast and simple readout signal from multi-well plates is essential. To achieve this goal, in this study, we expressed several genotypes of HLA in mammalian cells and determined their relative affinity with known antigen peptides. Based on the results, satisfactory combinations of HLA and peptide were selected, and we established, for the first time, a live cell- and 96-well plate-based high throughput screening system for antigen presentation inhibitors targeting DR1 (DRB1*0101) and DR15 (DRB1*1501).

## Results

### Cell-surface expression of HLA-DR in HEK293 cells

In this study, we attempted to establish a transfected cell-based peptide binding assay system in 96-well plate. Over the past decades, through intensive research, the amino acid sequence of self-antigen peptides presented by particular HLA-DR associated with autoimmune diseases was identified^1^. However, the relative strength of interaction between respective antigen peptide and HLA molecules is not clear. Therefore, we first measured the binding affinity of several epitope peptides to a selected set of HLA molecules expressed in HEK293T cells after transient transfection with respective HLA gene.

**Figure 1:**
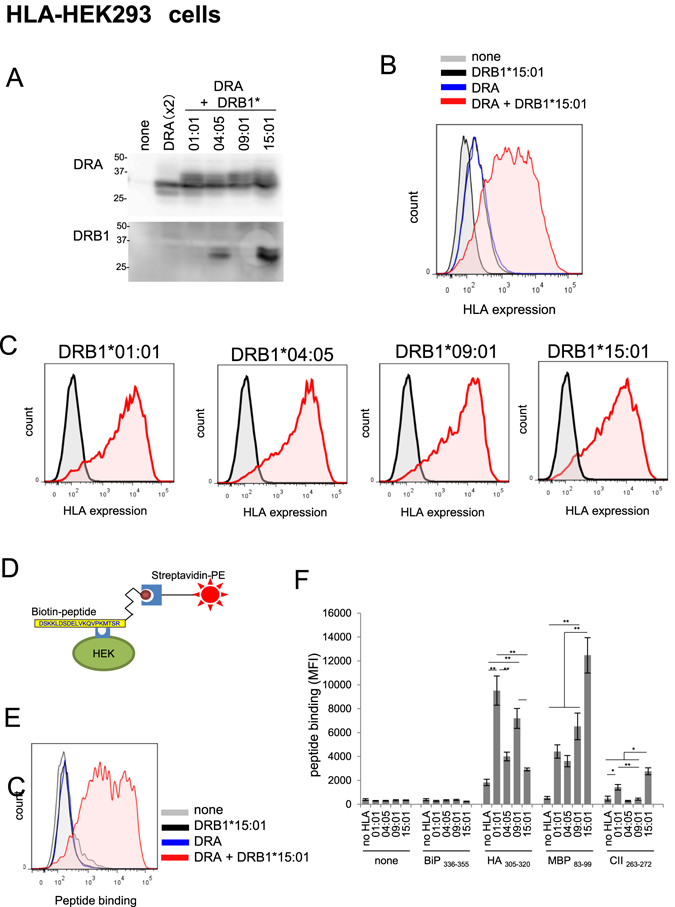
Expression of HLA molecules and binding to various antigen peptides in HEK293T cells. (**A**) Western blot analysis of the expression levels of HLA with anti-DRA or DRB1 antibody. (**B**) FACS analysis of cell-surface expression of DRA molecules after transfection with vector for DRA*0101, DRB1*15:01, or the combination of both. (**C**) FACS analysis of the expression levels of cell-surface HLA molecules in various co-transfected HEK293T cells. HEK293T cells were transfected with a plasmid vector for DRA*01:01 alone or in combination with the indicated expression vector for DRB1, and HLA protein expression levels in the Triton X-100-soluble fraction (**A**) or intact cell surface (**B** and **C**) was analysed 24 h later. The grey peak and pink peak represent untransfected control cells and respective DRB1 transfected cells, respectively. For each data, one set of representative result is shown. (**D**) Scheme of FACS analysis of peptide binding to cell-surface HLA molecules. HLA-expressing cells were incubated with biotinylated antigen peptide, and HLA-bound peptides were probed with SA-PE, followed by FACS analysis. (**E**) Specificity of peptide binding. HEK293T cells transfected as in (**B**) were incubated with biotinylated MBP83-99 (30 µM) for 5 h, and bound peptides were analysed by FACS. (**F**) Summary of relative binding affinity of HLA-DR and epitope peptides. Peptide binding assay was carried out in various HLA-expressing cells as in (**E**) (see, Supplementary Figs 1 and 2). Mean fluorescence intensity was plotted as mean ± inter-assay deviation expressed as SEM from 3 to 6 independent experiments. *P < 0.05 and **P < 0.01.

In the first series of experiment, we assessed whether 1:1 co-expression of DRA and DRB1 molecules could form a stable and functional α/β heterodimer on the cell surface of non-APC HEK293T cells. HEK293T cells were transiently transfected with the DRA*01:01 expression vector alone or in combination with either DRB1*01:01, DRB1*04:05, DRB1*09:01, or DRB1*15:01, and the levels of HLA α- and β-chain in each combination were analysed by western blotting. As shown in Fig. 1A, co-expression of any DRB1 gene with DRA resulted in almost comparable levels of DRA protein. Interestingly, however, expression of DRA alone, although the DRA vector was transfected twice to mimic the co-transfection group showed significantly lower DRA levels, suggesting stabilisation of the HLA molecules by heterodimer formation^18^. We also assessed the relative expression levels of DRB1 protein. However, this was not possible due to the isotype-dependency of anti-DRB1 antibodies. For example, using an antibody from Abcam (ab89315, Cambridge, MA, USA), only DRB1*04:05 and DRB1*15:01 could be detected (Fig. 1A, lower blot).

Next, we confirmed cell-surface expression of HLA molecules by FACS (Fig. 1B). The anti-HLA-DR antibody (clone L243) used in this experiment was conformation-specific and detect α-chain when α/β heterodimer are properly formed^23^. Expression of either DRA or DRB1*15:01 resulted in a marginal increase in HLA immunofluorescence. In contrast, 1:1 co-expression of DRA and DRB1*15:01 brought about significant surface staining of HLA, consistent with a previous study demonstrating that both α and β chains are necessary for cell-surface expression^18^. Similarly, requirement for co-expression of DRB1 and DRA for cell-surface expression was also evident in other DRB1 genotypes, and almost the same levels of cell-surface HLA expression was observed for any DRB1 genotypes tested (Fig. 1C).

### Binding affinity of antigen peptide to HLA molecules on HEK293T cells

Next, we examined whether the cell-surface-expressed HLA-DR molecules in HEK293T cells could accommodate exogenously added epitope peptides using a combination of HLA-DRB1*15:01 expressing cells and biotinylated MBP83-99 peptide (Fig. 1D). No peptide binding was observed when MBP83-99 peptide was included in cells transfected with either DRA*01:01 or DRB1*15:01 expression vector alone (Fig. 1E). In contrast, extensive binding of MBP83-99 was observed when cells expressed both DRA*01:01 and DRB1*15:01, a condition which resulted in the expression of a proper conformational α/β heterodimer. This result demonstrates that ectopically expressed HLA could load exogenously added epitope peptide.

To determine the genotype specificity and relative affinity of known epitope peptides, MBP83-99 and BiP336-355 peptide loading assays were performed on cells expressing DRA*01:01 plus various HLA-DRB1 (Supplemental Fig. 1A). No loading of either peptide was observed in control (non-transfected) HEK293T cells. In contrast, significant MBP83-99 binding was observed for any combinations of HLA heterodimer expressing cells, including DRB1*01:01, DRB1*04:05, DRB1*09:01, or DRB1*15:01. However, the extent of peptide loading, which is evaluated by the mean fluorescence intensity (MFI), was the highest in HLA-DR15-expressing cells among other cells (Supplemental Fig. 1A). BiP336-355 peptide was recently identified as a self-antigen presented by DR4 (DRB1*04:05) and is considered a causative factor for RA^24^. The peptide could bind to DR4-expressing HEK293T cells, although very slightly, but not to other DR-expressing cells. However, we could not observe a further increase in BiP336-355 binding to DR4 in terms of MFI value even though the incubation period was extended to 20 h (data not shown).

Next, the binding specificity of CII263-272 and HA305-320 peptides was assessed (Supplemental Fig. 1B). Significant binding of CII263-272 was detected in DR1 (DRB1*0101)- and DR15 (DRB1*15:01)-expressing cells although the MFI was far less than that for MBP83-99 binding (Fig. 1F). HA305-320 has been reported to bind to almost all genotypes of DRB1 molecules^25^. Although a significant level of apparent binding of HA305-320 was observed in non-transfected control cells, the extent of HA305-320 binding did increase in any genotypes of DRB1-expressing cells, with DR1 (DRB1*01:01) showing the highest level (Fig. 1F and Supplemental Fig. 1B). These results demonstrate that HLA-DR expressed in non-APC can present exogenously added antigen peptides in a genotype-specific manner.

### Establishment of stable cell lines expressing HLA-DRB1

Peptide binding experiments with HEK293T cells verified the genotype specificity in HLA-peptide binding as well as their binding strength. However, HEK293T cells detach easily from the culture matrix, which makes it difficult to remove unbound biotinylated peptides from bound ones on the attaching cells in the wells by washing. For this reason, we chose murine 3T3 fibroblast cells for the generation of stable HLA-DR-expressing cell lines. Using a lentivirus vector system, DRA*01:01 and each DRB1 vectors were introduced into the cells at 1:1 ratio, and cell-surface HLA-DR-expressing cells were enriched by a cell sorter (BD Aria, BD Biosciences, San Jose, CA, USA).

**Figure 2:**
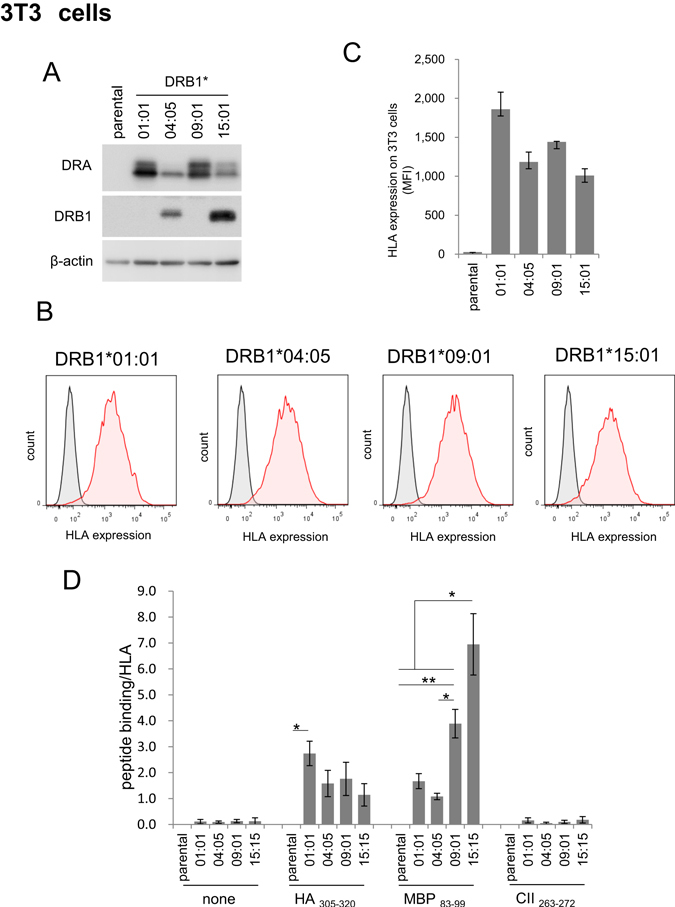
Stable expression of HLA molecules and binding to various antigen peptides in murine 3T3 cells. (**A**) Western blot analysis of HLA-DR expression levels in various HLA-expressing 3T3 cells. (**B,C**) FACS analysis of cell-surface expression of HLA-DR molecules in 3T3 cells. The grey peak and pink peak represent untransfected control cells and respective DRB1 transfected cells, respectively. The graph shown is a summary of the FACS results of two independent measurements and expressed as mean ± range (**C**). Western blotting and peptide binding assay were performed, and one representative result is shown. (**D**) Summary of the relative binding affinity of various HLA-DR and antigen peptides. Peptide binding assay was carried out in various HLA-expressing 3T3 cells (Supplementary Fig. 2). For each peptide group, peptide binding/HLA was calculated by subtracting the MFI value for the parental 3T3 cells from individual MFI. Then, the net MFI values were divided by the respective MFI value for HLA expression levels (Fig. 2C). The values are mean ± inter-assay deviation expressed as SEM of three independent experiments. Statistical significance is as follows: *P < 0.05 and **P < 0.01.

Expression levels of HLA-DR in these stable cell lines were analysed by western blotting and FACS analysis. In western blotting, anti-DRA antibody confirmed DRA protein expression in respective DRB1-expressing cells although its levels were different among the cell lines (Fig. 2A). The genotype-biased anti-DRB1 antibody (ab89315) again detected only DR4 (DRB1*04:05) and DR15 (DRB1*15:01), consistent with the results obtained by using HEK293T cells (Fig. 1A). FACS analysis also confirmed cell surface expression of HLA-DRA in these cells and was consistent with the results of western blot analysis (Fig. 2B and C).

Next, peptide loading capacity of these cell lines was assessed. Similar to HEK293T cells, a significant degree of apparent binding of HA305-320 was observed in control (parental) 3T3 cells, although the cells express no HLA-DR (Supplementary Fig. 2). Compared to the parental cells, however, HA305-320 peptide binding increased in cells expressing HLA, especially DR1. In contrast, MBP83-99 did not appreciably bind to parental cells, but potently bound to HLA expressing cells, particularly, DR15-expressing cells, consistent with the results obtained with HEK293T cells. Although very weakly, CII263-272 could bind to DR1-expressing cells, but not at all to other DR-expressing cells.

Overall, there results verify that HLA-DR molecules on the cell surface of these stable cell lines are functional. Since the cell surface expression levels of HLA were different among the cell lines (Fig. 2C), the relative extent of binding was normalised by subtracting HLA-independent peptide binding (MFI for parental 3T3 cells), followed by dividing by the individual expression level of cell-surface HLA (MFI for individual cell line) (Fig. 2D). The resultant profile was almost identical with that obtained in HEK293 cells (Fig. 1F), demonstrating that the specificity of peptide-HLA binding was determined by HLA genotype rather than the type of host cells.

### HLA-peptide binding assay in 96-well plate

The FACS results with 3T3 cells demonstrated that, among the exogenously applied peptides, only HA305-320 and MBP83-99 could be loaded onto HLA to a sufficient level in an allele-dependent manner; HA305-320 to DR1 (DRB1*01:01) and MBP83-99 to DR15 (DRB1*15:01). Therefore, using a combination of MBP83-99 and DR15-expressing 3T3 cells, we optimised the assay conditions for 96-well plates.

Initially, HLA-bound biotinylated MBP peptides were probed with a saturated concentration of SA-β-gal, and colour development was conducted using the colorimetric *o*-nitrophenyl-β-galactoside (ONPG) as a substrate. Under the assay conditions, however, we could not observe prominent genotype-selectivity in peptide binding as observed in the same cells using FACS analysis. For instance, at 300-fold dilution of SA-β-gal with ONPG, MBP83-99 (5 µM) binding to DR15 was at most 2-fold over that to parental cells (Supplemental Fig. 3). Eliminating cell fixation with glutaraldehyde did not improve the results (data not shown). Therefore, we serially decreased the concentration of SA-β-gal from a 300-fold dilution to up to a 10,000-fold dilution, and employed a highly fluorogenic β-galactosidase substrate, 4-methylumbelliferyl β-D-galactopyranoside (4MUG), for the detection of minute levels of β-gal activity (Supplemental Fig. 4A to D). Although the fluorescence signal decreased with decreasing concentrations of SA-β-gal (Supplemental Fig. 4E), at 3,000-fold dilution, an almost 6-fold maximum difference in fluorescence signal for MBP83-99 (5 µM) binding over the parental cells was observed (Supplemental Fig. 4F). Therefore, we chose 3,000 -﻿fold dilution with 4MUG for the subsequent experiments in 96 well plates.

**Figure 3:**
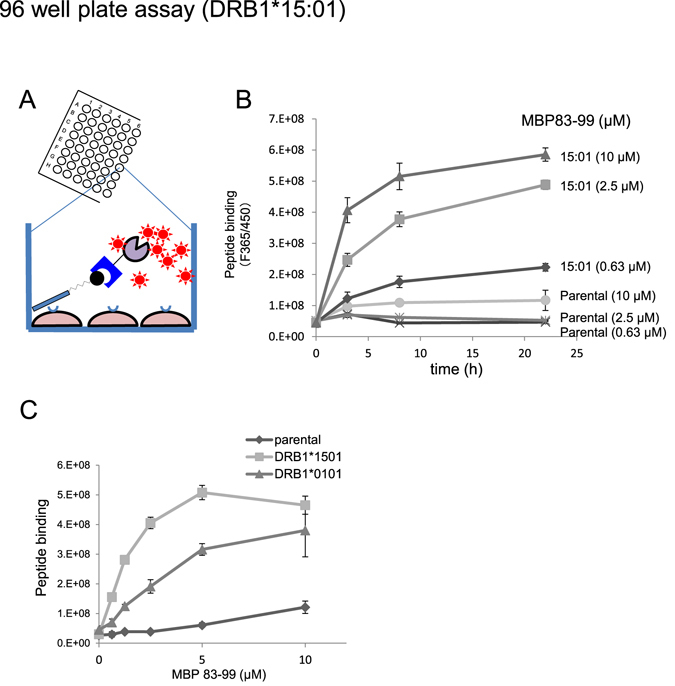
Detection and screening system of antigen peptide binding to HLA on 3T3 cells in 96 well plate. (**A**) Scheme of the detection of HLA-bound peptides on cells in 96 well plates. HLA expressing cells are incubated with biotinylated antigen peptides, and bound peptides are probed with SA-β-gal, followed by enzyme reaction with chromogenic (ONPG) or fluorogenic substrate (4MUG). (**B**) Detection of HLA-bound peptides on 3T3 cells using fluorogenic substrate (4MUG) in 96 well plates. Two 96-well plates of DR15-expressing cells were incubated with MBP83-99 for 6 h, fixed with glutaraldehyde, and subjected to blocking with a blocking solution as detailed in the methods section. Thereafter, biotinylated peptides in one plate were probed with low concentration of SA-β-gal (3,000-fold dilution) and detected by using 4MUG. The other plate was analysed using a chromogenic substrate (ONPG) in Supplementary Fig. 3. (*C*) Time and concentration dependence of MBP83-99 binding to DR15-expressing 3T3 cells. The assay was conducted as in (**B**). In either figure, values shown are mean ± intra-assay deviation expressed as SD from 3 wells in one set of representative experiments.

Using this 96-well plate assay procedure, we measured the time course and peptide concentration dependency of peptide loading in HLA-expressing 3T3 cells. MBP83-99 can be loaded onto DR15-expressing 3T3 cells in a time and concentration dependent manner, but not significantly onto parental 3T3 cells. Almost plateau level of loading could be attained in 8 h for each peptide concentration (Fig. 3B). Therefore, detailed concentration dependence of MBP83-99 loading to DR15 (DRB1*1501)-expressing cells was measured after 8-h incubation. MBP83-99 loading reached a plateau at above 5 µM of the peptide (Fig. 3C). Assuming that the concentrations of free MBP peptide in the wells at equilibrium are the same as the initial concentrations (i.e. the amount of HLA-bound peptide was negligible compared to the total amounts of the peptide in the wells), dissociation constant (Kd) was estimated. Analysis of the data from Fig. 3C by double reciprocal plot and Scatchard plot calculated the apparent Kd values to be 2 and 3 µM, respectively (Supplemental Fig. 5A and B).

As HA305-320 could effectively be loaded onto DR1-expressing cells (Fig. 1F and Fig. 2D), we also measured HA305-320 loading to DR1-expressing 3T3 cells (Supplemental Fig. 6A). The time course study revealed that HA305-320 binding reached a maximum level at 8 h, and then gradually decreased. However, unlike MBP83-99, but consistent with FACS analysis, a significant amount of HA305-320 binding was observed in the parental cells as well as in DR1-expressing cells. The involvement of HLA-independent binding signal (binding to parental cells) exceeded 80% of the entire signal obtained from DR1-expressing cells (supplemental Fig. 6B). Therefore, we excluded HA305-320 as a binding partner of DR1 in further study.

Since DRB1*0101 is an important risk gene for RA, we sought alternative ways to establish an assay system. MBP83-99 also bound to DR1-expressing 3T3 cells. Thus, we addressed the feasibility of this peptide for DR1 (DRB1*0101) plate assay (Fig. 3C). DR1-expressing cells could capture MBP83-99 although to a lesser extent than DR15-cells could. Apparent Kd values for MBP83-99 binding to DR1 by the double reciprocal plot and by Scatchard plot were 10 µM and 14 µM, respectively (supplemental Fig. 5C and D). The difference in MBP83-99 binding at 5 µM between parental cells and DR1-expressing cells indicated that the involvement of HLA-independent binding in the apparent signal was <30% (Fig. 3C). Although the value was higher than that for DR15 of <10%, DR1-expressing 3T3 cells with MBP83-99 as well as the DR15 counterpart, present potential as a drug screening assay system.

### Validation of the 96-well plate HLA-peptide binding assay as a drug screening system

**Figure 4:**
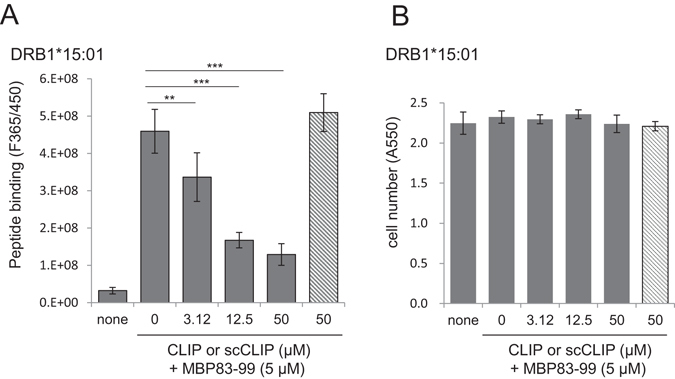
Effect of CLIP102-118 on the loading of MBP83-99 to HLA-expressing 3T3 cells in 96-well plates. (**A**) Effects of CLIP and scCLIP on MBP83-99 loading to DR15-expressing cells. (**B**) Cytotoxicity of CLIP. DR15-expressing 3T3 cells in 96-well plates were incubated without or with the indicated concentration of CLIP (filled bar) or scCLIP (hatched bar) for 1 h, and then exposed to MBP83-99 for 6 h. Bound peptides were detected as in (Fig. 3). After fluorescence measurement, the remaining cells in each well of were detected by crystal violet staining for cytotoxicity analysis. Values shown are mean ± intra-assay deviation expressed as SD from 3 to 6 wells in one representative result. **P < 0.01, ***P < 0.001.

Finally, using the combination of MBP83-99 and DR1- or DR15-expressing 3T3 cells, we validated our inhibitor screening system. CLIP102-118 is an endogenous peptide immobilised in the groove of nascent HLA molecules in the endoplasmic reticulum and is replaced with foreign peptides in the endosome^2^. It can act as a competitive inhibitor of peptide loading in cell-free assay system^26^. The reported Kd values range from 1 nM to 4 µM depending on the MHC/HLA genotypes and assay conditions^27, 28^. Here, we used CLIP as a model “test” compound in our assay system. Pre-incubation with CLIP102-118 for 1 h followed by exposure to MBP83-99 resulted in the apparent inhibition of MBP peptide loading in DR1- (Supplemental Fig. 6C) as well as in DR15-expressing 3T3 cells (Fig. 4A) with IC_50_ values being 8 µM and 9 µM, respectively. In contrast, a scramble version of the CLIP peptide (scCLIP) had no inhibitory effect on either HLA, demonstrating the specificity of the peptide interaction. Using the IC_50_ values, the apparent Kd values for CLIP and DR15 and DR1 were estimated to be 3 µM and 5 µM, respectively. Crystal violet staining of the assay plates after the binding assay showed no difference in the cell number (Fig. 4B and Supplemental Fig. 6D), demonstrating that the decline in the apparent peptide binding for both cells was due to binding inhibition rather than cell detachment by toxicity. Thus, our assay system can be used to select compounds capable of inhibiting peptide loading onto DR1 and DR15 while using the same plates, ruling out false positive results.

## Discussion

The major findings/achievements of our study are as follows. First, this is a first, transfected cells- and 96-well plate-based high-throughput screening system of inhibitor compounds of antigen presentation. Secondly, we showed that, while some reported antigen peptides (MBP83-99) can be easily loaded onto HLA on the cell surface after exogenous addition, others (BiP336-355 and CII263-272) could not. Third, the profiles of the intensity of peptide loading to HLA were almost the same regardless of the types of cells used for expression (HEK293T cells *vs*. 3T3 cells).

Interaction of antigen peptides with a particular genotype of HLA class II or MHC class II molecules has been studied mostly by using recombinant or affinity-purified HLA molecules in cell-free assay systems^14, 16, 17, 21, 24, 29–31^. In some cases, cell-based analyses were also conducted, in which immortalised homozygous B cell lines were used, although they still express genetically disparate classes of HLAs^32, 33^. Alternatively, as in this study, a particular allele of HLA was focused and expressed in mammalian non-APC by transfection. However, detection of bound peptide on HLAs in these cells was generally conducted by FACS^21, 34, 35^ and/or by biological responses of co-cultured antigen-specific T cell hybridomas, including IL-2 secretion and ^3^H-thymidine incorporation^21, 22, 33, 35, 36^. For cell-based HLA-peptide binding assays, there is no report that utilises 96-well plates in combination with simple read-out methodologies such as fluorescence reading or enzyme activity assays, suggesting a hidden technical obstacle to achieve the goal.

In this study, we successfully detected HLA-bound peptide on the cell surface in 96-well plates. We believe that one of the keys to our success is the concentration of SA-β-gal. Although our FACS analysis clearly unveiled genotype dependency of peptide-HLA interaction in 3T3 cells as well as in HEK293T cells (Figs 1F and 2D), in 96-well plate assay, we initially failed to observe genotype dependency in the same cells (Supplemental Fig. 3). By limiting the concentration of SA-β-gal to 1/10 of the initial conditions with the use of the fluorogenic substrate, 4MUG, we could successfully unveil the genotype dependence of peptide-HLA binding in 96-well plate assay (supplemental Fig. 4). The difference may be attributed to the selective localisation of HLA molecules in the membrane raft domains of the plasma membrane^2, 37^. That is, even if all the HLA molecules have captured biotinylated peptides, steric hindrance due to the large molecular size of SA-β-gal molecules (1,080 kDa) may prevent themselves from detecting all the HLA-bound peptides. By decreasing SA-β-gal concentration to a non-saturated level, biotinylated peptides could be detected proportionally to their density on the cell surface in exchange for curtailing maximum available signal output. In FACS analysis, by contrast, the reaction with SA-PE of biotinylated peptide on cells was carried out in the cell-suspension state after cells were mechanistically detached from the matrix. Cell detachment disrupts the actin cytoskeleton, which, in turn, disorganises the membrane raft structures^38, 39^, allowing dispersion of HLA molecules throughout cell surface; a condition favouring the detection of biotinylated peptides by SA-PE.

There are potential advantages and disadvantages of the cell-based screening system compared with protein-based cell-free counterparts. The most important advantage is that the cell-based assay possibly represents more physiological conditions of APCs than cell-free assays. Secondly, in cell-free assay, peptide binding is sometimes remarkably slow, with the equilibrium being reached after incubation in the order of days^11, 15, 33, 40^, while, in our assay, both MBP83-99 and HA305–320 loading almost reached a plateau in 8 h. In this regard, in cell-free assays, peptide binding reaction was carried out at low pH to mimic endosome environment, as well as to facilitate peptide loading^15, 33^. Third, cell-free studies revealed that HLA proteins transit between a receptive and a non-receptive conformation^41^. Therefore, if this transition is more dynamic in live cells, a structurally broader spectrum of inhibitor compounds can be selected with cell-based assay. Lastly, our cell-based assay can also measure the toxicity of test compounds, which is, on one hand, advantageous in terms of information on the nature of test compounds while, on the other hand, may be disadvantageous by imposing a limitation on the assessable test compounds.

From this study, we suggest that the following three factors seemingly affect peptide-HLA binding in non-APCs: i) the expression level of HLA on the cell surface, ii) the affinity of peptide to target HLA, and iii) the mechanism of peptide loading onto HLA. Although genotype-dependent binding profiles of MBP83-99 and HA305-320 were almost the same between HEK293T and 3T3 cells, the binding intensities of both peptides in 3T3 cells was far less than those in HEK293T cells (Supplemental Fig. 1 *vs*. Supplemental Fig. 2). The difference can simply be attributed to the expression levels of HLAs as FACS analysis of HLA expression showed that the expression in 3T3 cells was one order of magnitude lower than that in HEK293T cells (Figs 1C *vs*. 2B). The relatively low expression levels, along with the lower affinity of the peptide compared to that of MBP83-99, could also account for the lack of binding of CII263-272 in 3T3 cells. Alternatively, the mechanisms of loading of individual peptide are different. In APCs, although a portion of empty HLA molecules on the plasma membranes can directly capture foreign peptides^41^, generally peptide loading takes place in acidic endosomal compartments with the assistance of the chaperone molecules, HLA-DM^2, 41^. Loading of CII263-272 and BiP336-355 may need an endosomal chaperone molecule like HLA-DM in a cell-type dependent manner.

HA305-320 binds to every genotype of HLA-DRs^25^ and is often used as a reporter binding partner of recombinant HLAs in cell-free assays^24, 29^. In our FACS analysis, however, this peptide bound to the cell surface independently of HLA, especially avidly in 3T3 cells, but not so severely in HEK293T cells. This high basal binding prevented HLA-expressing 3T3 cells from further development into 96-well assay. Thus, if the HLA expression level was high as in HEK293T cells, non-specific cell surface binding became negligible. However, if the HLA expression level was low as in 3T3 cells, the high background could mask specific binding. For the assay with sticky peptide, HLA expression level must be high.

Peptide vaccination intended to activate HLA class II-restricted CD4 T cells garnered the attention in relation with cancer therapy^42–44^, and several “enhancer” compounds for peptide loading onto HLA have been reported^21, 33, 36, 41^. It is possible to identify HLA-interacting sequences from tumour antigens for vaccine design through competition assay as verified for CLIP (Fig. 4). Additionally, our assay system can identify enhancer compounds for peptide vaccine loading onto HLA. Association of HLA genotypes with idiosyncratic drug toxicities (IDTs) has been suggested^45^. Our assay system can also be used to conduct risk assessment of new drugs.

In conclusion, we established the first cell- and 96-well-based screening system for antigen peptide loading modulator. The assay system can discriminate the efficiency of test compounds from their toxicity in the same plates. The choice of peptide, read-out system, and the expression levels of HLA molecules are all critical determinant in the successful construction of the assay system. Our assay system could have the potential for broad applications, including designing of peptide vaccines.

## Methods

### Reagents and cells

β-galactosidase-conjugated streptavidin (SA-β-gal) was purchased from Life Technologies Inc (Carlsbad, CA, USA) and reconstituted with distilled water to 2 mg/mL according to the manufacturer’s instructions. X-tremeGENE HP, 4-Methylumbelliferyl β-D-galactopyranoside (4MUG), and anti-β-actin antibody were purchased from Sigma (St Louis, MO, USA). Crystal violet, o-nitrophenyl-β-D-galactopyranoside (ONPG), and Sepasol were purchased from Nacalai Tesque (Kyoto, Japan). APC-labelled anti-HLA-DRA antibody (307609) and Phycoerythrin-labelled streptavidin (SA-PE) were from Biolegend (San Diego, CA, USA). Anti-HLA-DRA antibody (ab92511) and anti-HLA-DRB1 antibody (ab89315) were purchased from Abcam (Cambridge, MA, USA). A set of plasmids for lentivirus expression vector system, including human immunodeficiency virus type 1 (HIV-1)-based CSII-MCS-EGFP, pCAG-HIVgp, and pCMV-VSV-G-RSV-Rev, was obtained from RIKEN (Wako, Osaka, Japan).

The peptide corresponding Class II-associated invariant chain peptide (CLIP) 102–118, KPVSKMRMATPLLMQAL, and its scramble peptide (scCLIP), LMAKARQKVLMPSTMLP, and the following biotinylated peptides were synthesised by Biologica Co. (Nagoya, Japan) with a ε-aminocaproic acid linker between the biotin moiety and N-terminal of peptide: influenza hemagglutinin (HA) 305–320, ACPKYVKQNTLKLATG; myelin basic protein (MBP) 83–99, ENPVVHFFKNIVTPRTP; immunoglobulin binding protein (BiP) 336–355, RSTMKPVQKVLEDSDLKKSD; and type II collagen (CII) 263–272, GIAGFKGEQGPKGEP. The purity of these peptides were >95%.

HEK293T and 3T3 cells kindly provided by Dr. Takehito Sato at Tokai University School of Medicine and were maintained in Dulbecco’s modified Eagle (DMEM) medium supplemented with 10% foetal calf serum and antibiotics.

### Cloning of HLA and construction of HLA expression vectors

The collection and use of human blood from healthy volunteers were approved by the Medical Research Ethical Committee of Tokai University (14I-26), and written informed consent was obtained from all participants. All the experiments were carried out in accordance with the relevant guidelines and regulations.

Peripheral blood mononuclear cells were separated from the blood using Vacutainer vacuum tube (BD Biosciences, San Jose, CA, USA). Genomic DNA was isolated, and HLA genotype was determined by GenoDive Pharma, Inc. (Kanagawa, Japan). mRNA was isolated by using Sepasol (Nacalai Tesque), and cDNA library was prepared by using High-Capacity cDNA Reverse Transcription Kit (Thermo Fisher Scientific, Waltham, MA, USA) according to the respective manufacturer’s protocols. The open reading frame for DRA or DRB1 were flanked with Xho 1 and EcoR1 sites at 5′ and 3′ end, respectively, and was amplified by PCR (KOD plus, Toyobo, Japan). The DNA sequences of the primers are follows:

DRA-forward: 5′-ACGCTCGAGATGGCCATAAGTGGAGTCCC-3′

DRA-reverse: 5′-CGTGAATTCTTACAGAGGCCCCCTGCGTT-3′

DRB1-forward: 5′-ACGCTCGAGATGGTGTGTCTGAAGTTCCC-3′

DRB1-reverse: 5′-CGTGAATTCTCAGCTCAGGAATCCTGTTG-3′

The PCR fragments and CSII-MCS-EGFP plasmid were digested with Xho 1 and EcoR1, and the DRA or DRB1 fragments were ligated to the CSII plasmid. The plasmids were amplified in *E. coli* DH5α strain and purified by using Fast Gene Midi kit (NIPPON Genetics Co, Ltd, Tokyo, Japan) according to the manufacturer’s instructions. The DNA sequences for the HLA region of these vectors were confirmed by DNA sequencing.

### Transient transfection of HLA plasmids in HEK293T cells

HEK293T cells at sub-confluence in 6-well or 24-well plates were transfected with CSII-DRA1 plasmid or CSII-DRB1 plasmid or the combination of both using X-treme transfection reagents with the DNA to reagent ratio of 1 µg to 3 µL. Twenty-four hours after transfection, cells from 6 well plates were used for western blot analysis, and cells from 24 well plates for FACS analysis (peptide binding and cell-surface staining). Under the experimental conditions, the transfection efficiency in either plate, as assessed by GFP expression, was >90% under microscopic observation.

### Western blot analysis of HLA expression

Cells were lysed in a lysis buffer (0.5% (v/v) Triton X-100, 150 mM NaCl, 50 mM Tris-HCl (pH 7.4), protease inhibitor cocktail (Roche, Basel, Switzerland)) on ice and centrifuged at 10,000 × *g* for 10 min to obtain Triton X-soluble supernatants. Protein concentrations were determined by DC-protein assay kit (BioRad, Hercules, CA, USA), and equal amounts of proteins were separated by SDS-PAGE using 12.5% polyacrylamide gel and transferred onto PVDF membranes. HLA molecules on the membranes were probed with the appropriate primary antibody, followed by horseradish peroxidase (HRP)-conjugated secondary antibody. Chemiluminescence reaction was performed by using HRP substrate (Millipore, Billerica, MA, USA), and signal was recorded with an image analyzer (ATTO, Tokyo, Japan).

### Lentivirus-mediated transfection and establishment of HLA stably expressing cells

CSII-DRA1 plasmid or CSII-DRB1 plasmid, along with accessory plasmids pCAG-HIVgp and pCMV-VSV-G-RSV-Rev, was transfected into HEK293T cells at sub-confluence in 10 cm dishes using calcium-phosphate method^46^. Culture medium was collected at 48 h after transfection, and virus in the medium were concentrated by ultracentrifugation (VIVASPIN 20, Sartorius). 3T3 cells in 24 well plates were simultaneously infected with lentivirus vectors for DRA and DRB1 at 1:1 ratio (each MOI = 10). Ten days after infection, HLA-surface-expressing cell were labelled with APC-conjugated anti-HLA-DRA antibody and enriched by FACS Aria (BD Biosciences).

### Peptide binding assay and assessment of cell-surface HLA expression levels with FACS

HLA-expressing HEK293T cells or 3T3 cells in 24 well plates (about 2 × 10^5^ cells/well) were incubated with biotinylated peptides in 0.3 mL of medium for the indicated time period. Cells were detached from the wells by pipetting in PBS (for HKE293T cells) or 5 mM EDTA/PBS (for 3T3 cells), washed with 0.1% BSA/PBS, and suspended in 50 µL of the same medium. Bound peptides on the cells were probed with PE-conjugated streptavidin (×200, BioLegend) for 30 min, and were analysed by FACS (Verse, BD Biosciences). For detection of cell-surface HLA, cell suspensions in 0.1% BSA/PBS prepared as described above were incubated with APC-labelled anti-HLA-DR antibody (×100, BioLegend 307629) and analysed by FACS.

### Peptide binding assay using 96 well plates and plate reader

Unless otherwise described, the assay was conducted as follows. HLA-expressing cells were seeded in gelatin-pre-coated 96 well plates (about 3 × 10^4^ cells/well), and were exposed to the appropriate biotinylated peptide in the culture medium. After incubation for the indicated period, cells were washed with PBS and immobilised with 0.1% (v/v) glutaraldehyde (GA) in PBS for 5 min at room temperature, and blocked for 30 min with a blocking reagent (0.1% BSA, 0.2% gelatin, 0.75% glycine, 0.01% NaN_3_ in PBS)^47^. The cells were then incubated with SA-β-gal in 0.1% BSA/PBS (×3,000) for 30 min. After washing the wells three times with 0.1%BSA/PBS, the enzyme reaction was performed with 100 µL of substrate solution (100 µM 4MUG, 1.5 mM MgCl_2_, 5 mM 2-mercaptoethanol in PBS) at 30 °C for 30 min. The reaction was stopped by addition of 100 µL of 0.5 M Na_2_CO_3_, and fluorescence intensity (Ex: 365 nm, Em: 450 nm) was measured by using a plate reader (Spectra Max plus, Molecular Devices, Sunnyvale, CA, USA). Wells devoid of cells and incubation with biotinylated peptide, but otherwise treated by using the same experimental procedure, were set as blank, and the mean fluorescence value for the wells was subtracted. The apparent Kd values for the binding of MBP83-99 and DR1 (DRB1*01:01) or DR15 (DRB1*15:01) was calculated by double reciprocal plot (1/[P-HLA] = Kd/[HLA]_total_ [P] + 1/[HLA]_total_; where P represents peptide) or Scatchard plot ([P-HLA]/[P] = [HLA]_total_/Kd − [P-HLA]/Kd) after subtracting values for HLA-independent peptide binding (parental 3T3 cells) from the respective cells in a peptide concentration dependency experiment.

In some experiments, β-galactosidase activity was measured with 100 µL of substrate solution (2 mM ONPG, 2 mM MgCl_2_, 5 mM 2-mercaptoethanol in PBS) at 37 °C for 30 min, and absorbance at 405 nm was read on the plate reader.

For the assay of test compound, cells in 96-well plates were pre-incubated with test compounds in the medium for 1 h, followed by the addition of 10-fold concentrated appropriate biotinylated peptide. Detection of HLA-bound peptide was conducted by using 4MUG as described above. Apparent Kd values for CLIP were determined by using the following equation: Kd _for CLIP_ = IC_50_/(1 + [MBP]/Kd _for MBP_)^48^. The cytotoxic effect of test compound was assessed by crystal violet staining^49^. After fluorescence measurement, the plates were washed with PBS, and GA-fixed cells in the wells were stained with 100 µL of 0.5% (w/v) Crystal violet, 1% ethanol for 30 min, and washed three times with tap water. The stained cells were dissolved in 150 µL of 1% (w/v) sodium deoxycholate solution, and absorbance at 550 nm was measured in a plate reader (Spectra Max).

### Statistics

Experimental data were analysed by ANOVA followed by the Tukey test using GraphPad PRISM 5 (GraphPad, San Diego, CA, USA).

## Electronic supplementary material

Supplemental Figures
