# Supplementary material for: A cell-based high-throughput screening assay system for inhibitor compounds of antigen presentation by HLA class II molecule
Source: Sci Rep. 2017 Jul 28;7:6798. doi: 10.1038/s41598-017-07080-4 (PMC5533769; doi:10.1038/s41598-017-07080-4)
Supplement: Supplementary file 1 — Supplemental Figures [file 41598_2017_7080_MOESM1_ESM.pdf]

Supplementary Figures

A cell-based high-throughput screening assay system for inhibitor compounds of antigen presentation by HLA class II molecule

Nobuo Watanabe, Yusuke Suzuki, Takahisa Yonezu, Yuki Nakagawa, Takashi Shiina, Noriaki Hirayama, Sadaki Inokuchi, and Shigeaki Inoue

Supplemental Fig. 1

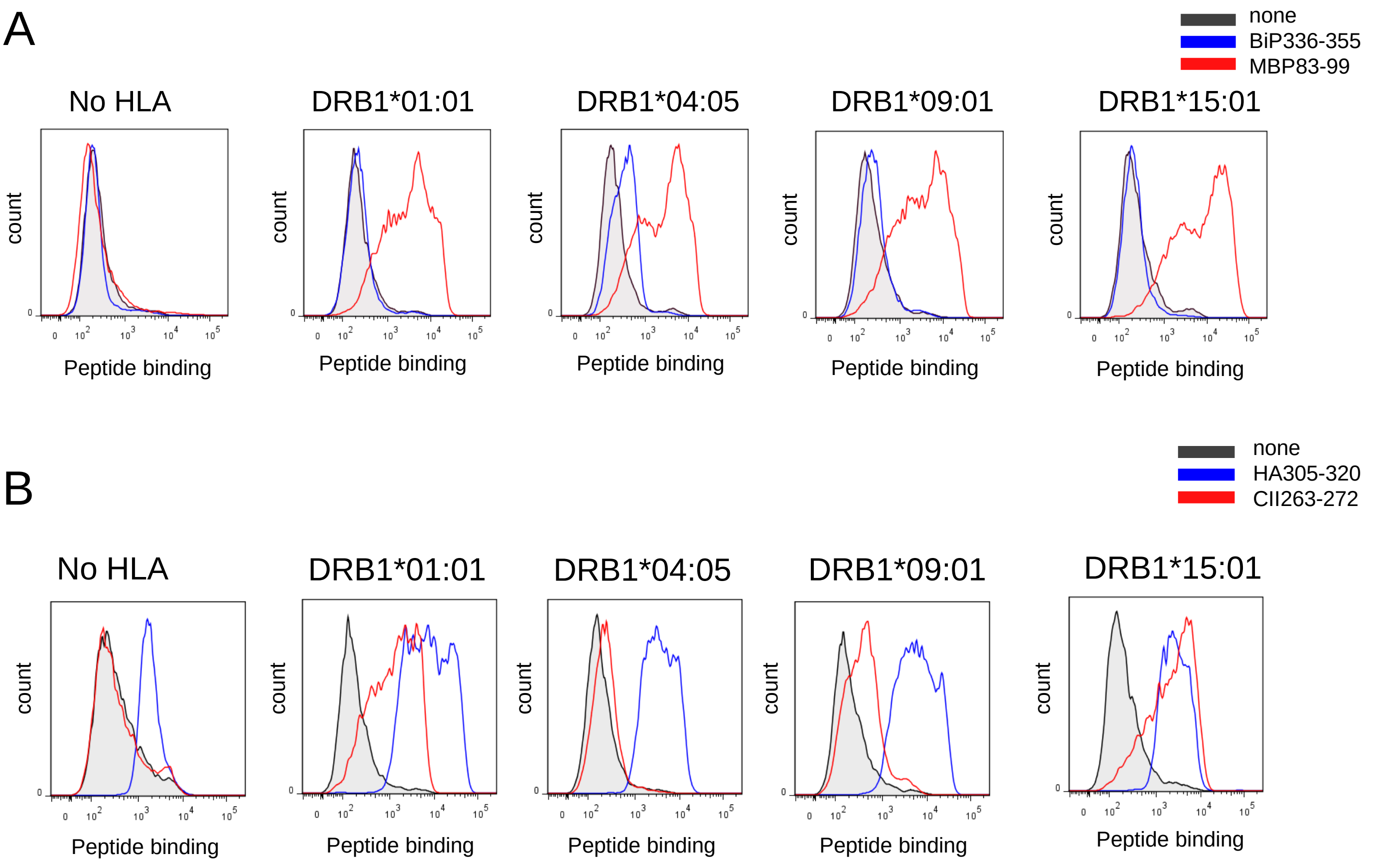

Supplemental Figure 1. FACS analysis of antigen peptide binding to various HLA molecules expressed in HEK293T cells.

DRA1 plus indicated genotype of DRB1 expression vector were co-transfected in HEK293 cells. The cells were then incubated with BiP336-355 or MBP83-99 peptide (A), or HA305-320 or CII263-272 peptide (B), each at 30 μM, for >5 h, and cell-bound biotinylated peptide was probed with SA-PE and analysed by FACS as in Fig. 1E. For each FACS data, one representative result is shown.

Supplemental Fig. 2

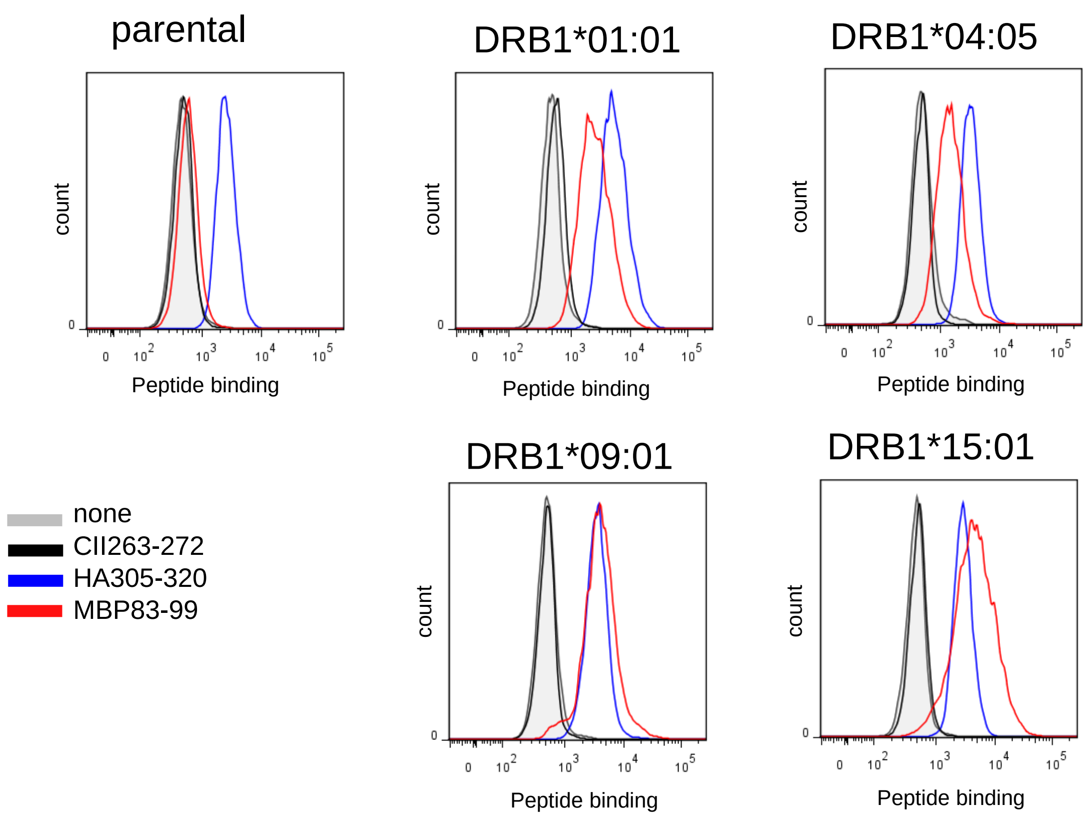

Supplemental Figure 2. FACS analysis of antigen peptide binding to various HLA in 3T3 cells. The indicated genotype of HLA-expressing 3T3 cells were incubated with 30 µM of MBP83-99, HA305-320, or CII263-272 peptide overnight, and cell-bound biotinylated peptide was probed with SA-PE and analysed by FACS as in Supplementary Fig. 1. One representative set of results is shown.

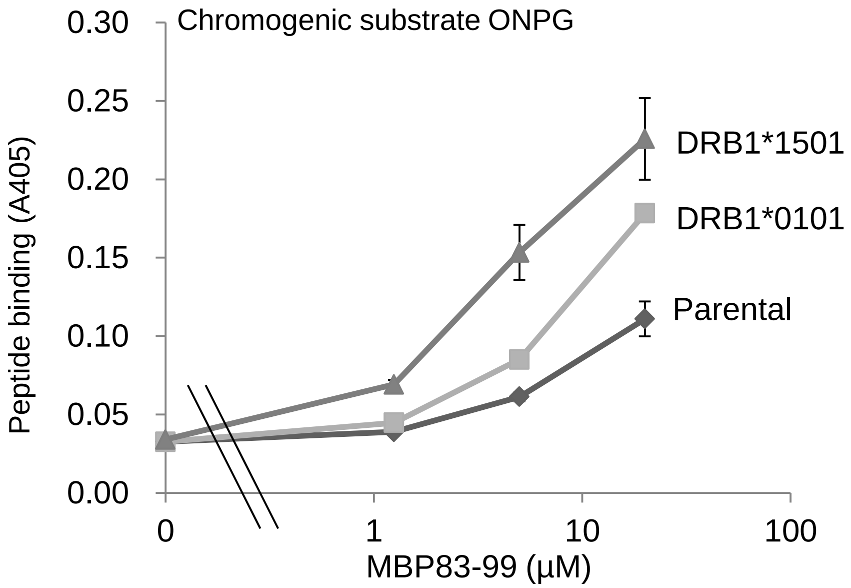

Supplemental Figure 3. Detection of HLA-bound peptides on 3T3 cells using a chromogenic substrate (ONPG) in 96 well plates. In this 96-well plate containing MBP83-99 exposed DR1- or DR15-expressing cells (as in Fig. 3C), the peptide was detected by using a high concentration of SA-β-gal (300-fold dilution) and ONPG. Values shown are mean ± intra-assay deviation expressed as SD from 3 wells in one set of representative experiments.

Supplemental Fig. 4

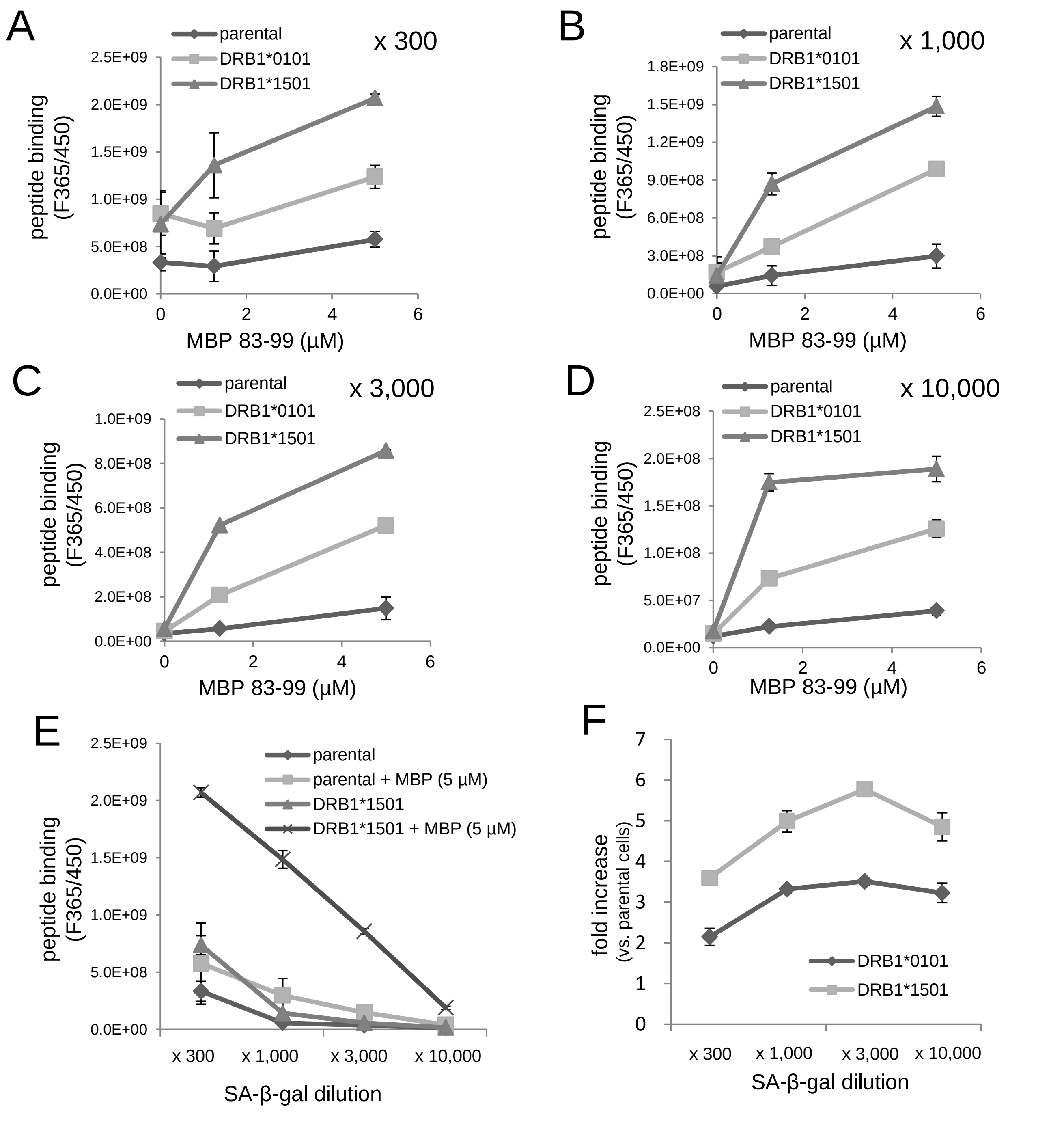

Supplemental Figure 4. Effect of concentrations of streptavidin-β-galactosidase on the detection of HLA-bound MBP83-99.

MBP83-99 peptide binding was carried out on 4 identical set of HLA-expressing 3T3 cells with 4MUG as a substrate, but with different dilution of SA-β-gal, as follows: (A) 300, (B) 1,000, (C) 3,000, (D) 10,000. In (E), the relationship between dilution of SA-β-gal and fluorescence intensity in parental and DR15 (DRB1*1501)-expressing cells with and without MBP83-99 (5 µM) binding from (A) to (D) was replotted. In (F), fold difference between parental and DR1- or DR15-expressing cells at 5 µM MBP83-99 binding. Assays were conducted as in Supplemental Fig. 3. In either figure, values shown are mean ± intra-assay deviation expressed as SD from 3 wells in one set of representative experiments.

Supplemental Fig. 5

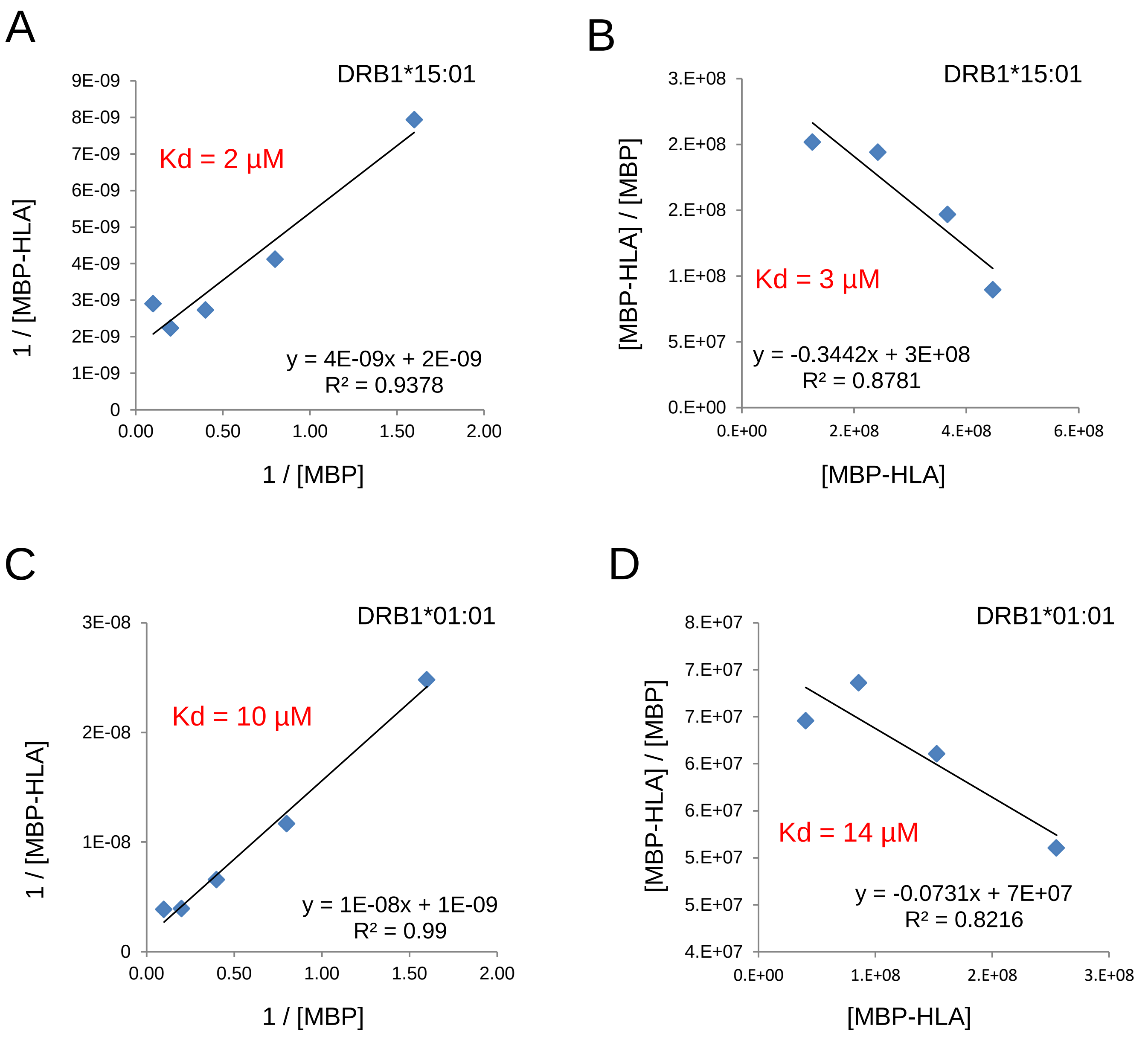

Supplemental Figure 5. Determination of apparent Kd values for MBP83-99 and DR1 or DR15

Double reciprocal plot analysis (A, C) of the binding of MBP83-99 to DR15 (DRB1*15:01) (A) or DR1 (DRB1*01:01) (C), or Scatchard plot analysis (B, D) of the binding of MBP83-99 to DR15 (DRB1*15:01) (B) or DR1 (DRB1*01:01). HLA-dependent binding was calculated by subtracting fluorescence intensity of parental cells from respective cells at each point (Fig. 3C).

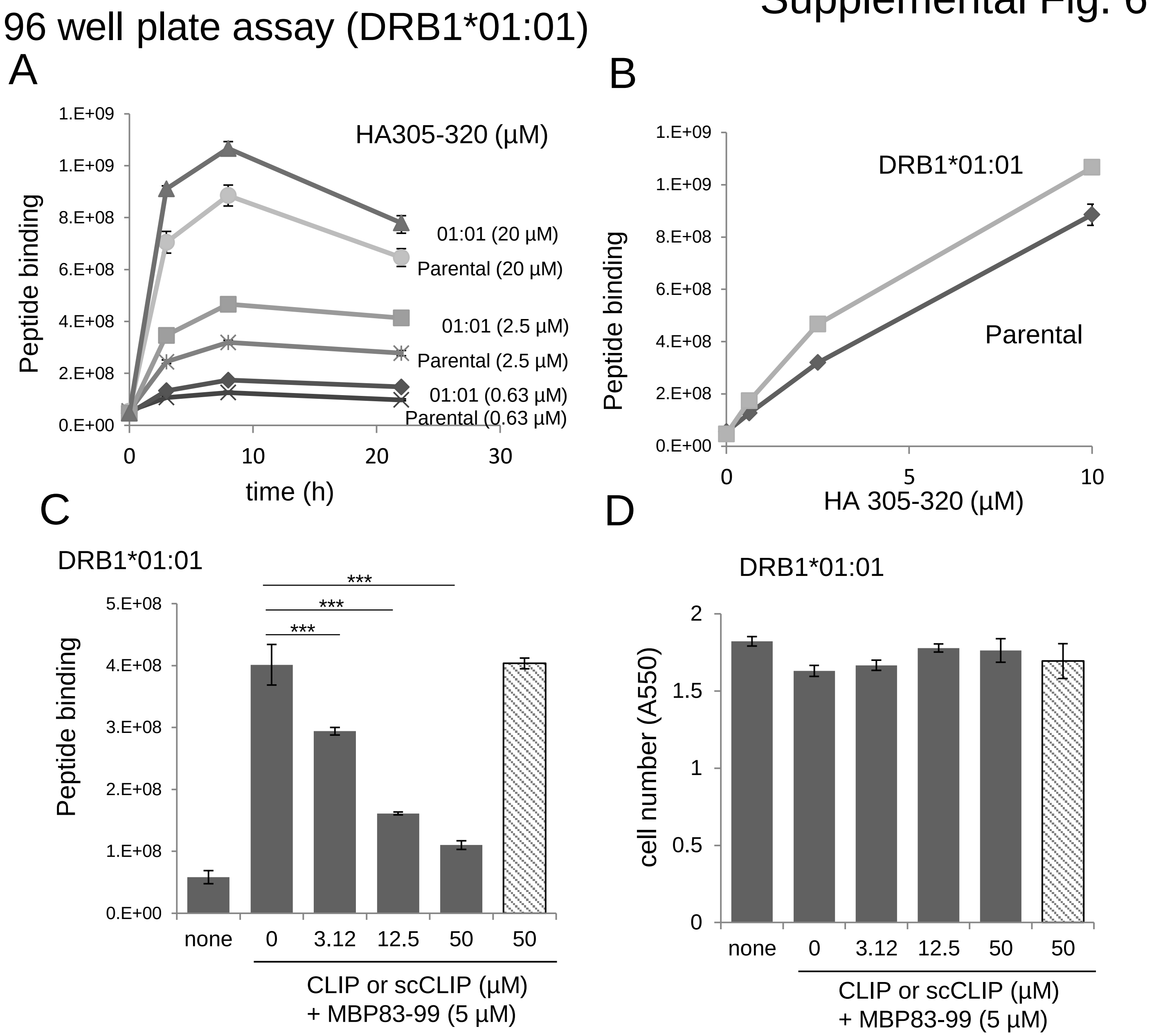

Supplemental Figure 6. Peptide binding assay in DR1 (DRB1*0101)-expressing 3T3 cells in 96 well plate. (A) Time and concentration dependence of HA305-320 binding to DR1-expressing 3T3 cells. The assay was conducted as in Fig. 3C. (B) Replotting of the concentration dependence of HA305-320 binding obtained at 8 h in (A). In either figure, values shown are mean ± intra-assay deviation expressed as SD from 3 wells in one set of representative experiments. (C) Effect of CLIP (filled bar) or scramble CLIP (scCLIP, hatched bar) on MBP83-99 loading to DR1-expressing cells. (D) Cytotoxicity of CLIP. DR1-expressing 3T3 cells in 96-well plates were incubated without or with 5 µM or 50 µM CLIP for 1 h, and then exposed to MBP83-99 for 6 h. Bound peptides were detected as in (Fig. 4). After fluorescence measurement, the remaining cells in each well were detected by crystal violet staining for cytotoxicity analysis. Values shown are mean ± intra-assay deviation expressed as SD from 3 to 6 wells in one representative result. ***, $P < 0.001$

Supplemental Fig. 7

Full-length blot

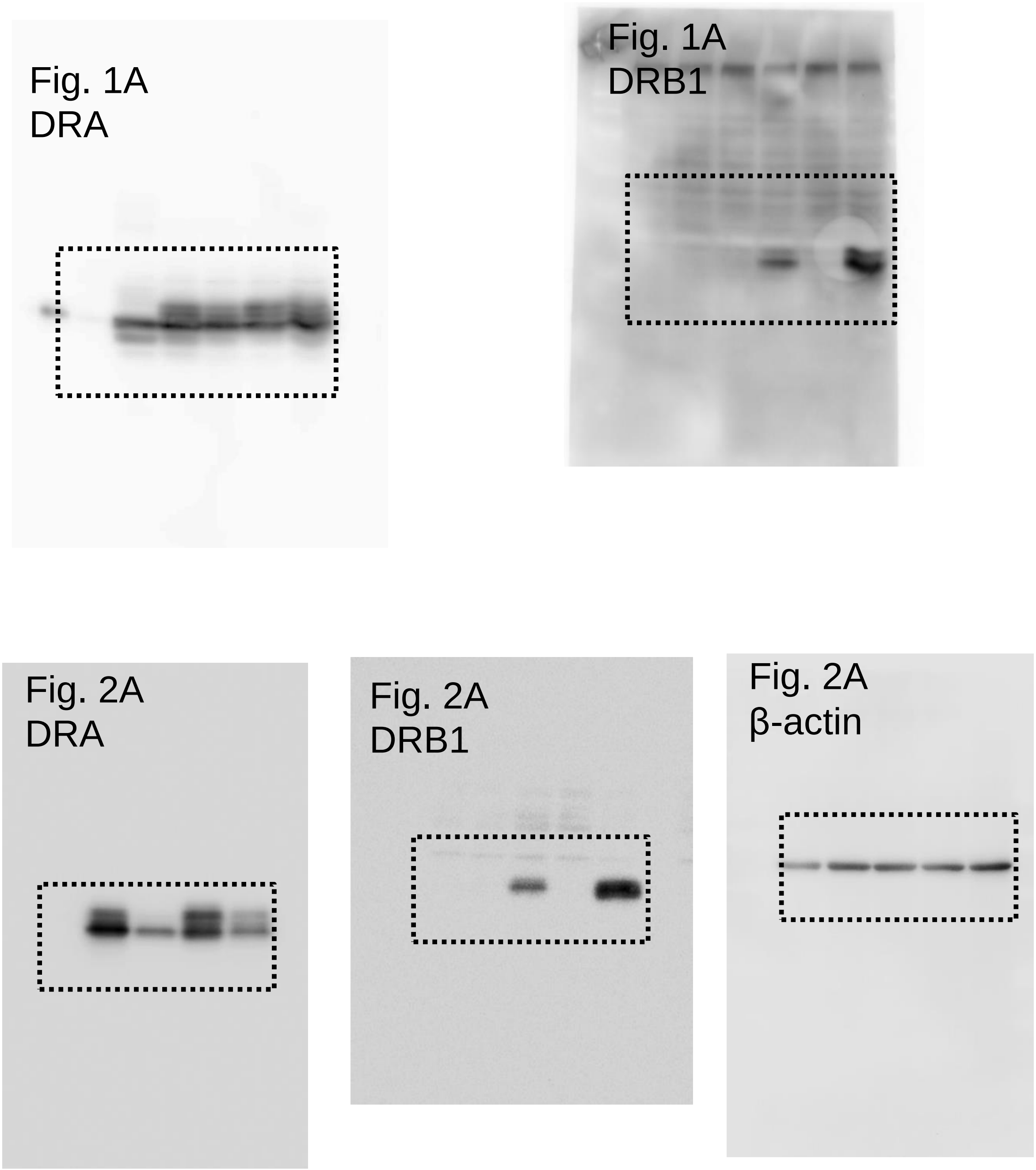

Supplemental Figure 7. Full-length blots for Figures 1A and Figure 2A
